# Autosomal Dominant Tubulointerstitial Kidney Disease with Adult Onset due to a Novel Renin Mutation Mapping in the Mature Protein

**DOI:** 10.1038/s41598-019-48014-6

**Published:** 2019-08-12

**Authors:** Céline Schaeffer, Claudia Izzi, Andrea Vettori, Elena Pasqualetto, Davide Cittaro, Dejan Lazarevic, Gianluca Caridi, Barbara Gnutti, Cinzia Mazza, Luca Jovine, Francesco Scolari, Luca Rampoldi

**Affiliations:** 1 Molecular Genetics of Renal Disorders, Division of Genetics and Cell Biology, IRCCS San Raffaele Scientific Institute, Milan, Italy; 2 Division of Nephrology and Dialysis, Department of Medical and Surgical Specialties, Radiological Sciences, and Public Health, University of Brescia and Montichiari Hospital, Brescia, Italy; 3 Prenatal Diagnosis Unit, Department of Obstetrics and Gynecology, ASST Spedali Civili, Brescia, Italy; 4 Department of Biology, University of Padova, Padova, Italy; 5 Department of Biotechnology, University of Verona, Verona, Italy; 6 Center for Translational Genomics and Bioinformatics, IRCCS San Raffaele Scientific Institute, Milan, Italy; 7 Laboratory of Molecular Nephrology, Istituto Giannina Gaslini IRCCS, Genoa, Italy; 8 Laboratory of Medical Genetics, Department of Pathology, ASST Spedali Civili, Brescia, Italy; 9 Department of Biosciences and Nutrition & Center for Innovative Medicine, Karolinska Institutet, Huddinge, Sweden

**Keywords:** Disease genetics, Interstitial nephritis, Interstitial nephritis, Mutation

## Abstract

Autosomal dominant tubulointerstitial kidney disease (ADTKD) is a genetically heterogeneous renal disorder leading to progressive loss of renal function. ADTKD-*REN* is due to rare mutations in renin, all localized in the protein leader peptide and affecting its co-translational insertion in the endoplasmic reticulum (ER). Through exome sequencing in an adult-onset ADTKD family we identified a new renin variant, p.L381P, mapping in the mature protein. To assess its pathogenicity, we combined genetic data, computational and predictive analysis and functional studies. The L381P substitution affects an evolutionary conserved residue, co-segregates with renal disease, is not found in population databases and is predicted to be deleterious by *in silico* tools and by structural modelling. Expression of the L381P variant leads to its ER retention and induction of the Unfolded Protein Response in cell models and to defective pronephros development in zebrafish. Our work shows that *REN* mutations outside of renin leader peptide can cause ADTKD and delineates an adult form of ADTKD-*REN*, a condition which has usually its onset in childhood. This has implications for the molecular diagnosis and the estimated prevalence of the disease and points at ER homeostasis as a common pathway affected in ADTKD-*REN*, and possibly more generally in ADTKD.

## Introduction

Autosomal Dominant Tubulointerstitial Kidney Disease (ADTKD) is a rare genetic disorder characterized by autosomal dominant transmission, progressive chronic kidney disease and renal tubulointerstitial fibrosis. To date, identified ADTKD-associated genes are *UMOD* (uromodulin, 16p12), *MUC1* (mucin-1, 1q22), *HNF1B* (HNF1-beta, 17q12), *REN* (renin, 1q32) and *SEC61A1* (alpha1-subunit of translocon 61, 3q21). Still, some of the families with ADTKD do not present mutation in the reported genes suggesting further genetic heterogeneity^1^.

The large majority of ADTKD patients are carriers of mutations in *UMOD* or *MUC1* genes^2,3^. ADTKD-*REN* (previously known as Familial Juvenile Hyperuricemic Nephropathy type 2 [FJHN2], MIM# 613092) represents a very rare condition, as so far 6 families have been described worldwide^4–7^. Although most of ADTKD characteristics including clinical, laboratory and histological findings are rather nonspecific, some features appear to be relatively distinctive for individual genetic forms. In particular, ADTKD-*REN* is characterized by hypoproliferative anemia with low hemoglobin concentration in the first decade of life and by childhood or adolescence onset of chronic kidney disease (CKD) and hyperuricemia due to decreased fractional excretion of urinary uric acid. The disease is also associated with bland urinary sediment, low plasma renin activity, mildly elevated serum potassium concentration, polyuria and slightly decreased blood pressure. Patients slowly progress to end-stage renal disease (ESRD) in the fourth to sixth decade of life^6,8^.

Renin is a secreted aspartyl protease that plays a key role in the renin–angiotensin system (RAS), an hormone system responsible of blood pressure and fluid balance regulation^9^. It is indeed the rate limiting factor in the RAS activation cascade by cleaving angiotensinogen to angiotensin I. Renin is synthesized as preprorenin that is translocated into the ER where it is processed to prorenin, its inactive precursor, by cleavage of the leader peptide. While prorenin has been found to be synthesized by many tissues^10^, mature renin is exclusively produced and secreted in the kidney by juxtaglomerular cells (JGCs). In JGCs prorenin is sorted from the Golgi to the constitutive or the regulated secretory pathway^11^ and can be directly secreted as prorenin or stored in secretory granules where it is converted into mature protein by proteolytic cleavage of the prosegment at a pair of basic amino acids (Lys65-Arg66) at the amino terminal part of the protein.

Mutations in *REN* were first described in autosomal recessive renal tubular dysgenesis (RTD [MIM# 267430]), a rare disease characterized by perinatal mortality and homozygosity or compound heterozygosity for loss-of-function *REN* mutations causing complete loss of renin synthesis^12^.

Instead, dominant mutations reported to date in ADTKD-*REN* families are all non-truncating, missense changes (W10R, L16R, W17R and C20R) or an in-frame deletion (L16del) mapping in the first exon of the gene and affecting residues within the protein leader peptide^4–7^. To date only three mutations (L16del, L16R, C20R) have been studied in transfected cells^4–6^. Such studies showed that protein co-translational insertion in the endoplasmic reticulum (ER) was impaired (L16R, C20R) or partly affected (L16del) and, at least for the L16del, this was shown to be associated with ER stress and activation of the Unfolded Protein Response (UPR)^4^.

In this study, we report a new ADTKD family in which we identified a novel renin mutation mapping in exon 10 of the gene, hence outside of the region where all previously identified mutations have been reported (exon 1)^4–7^. Affected family members presented adult-onset chronic tubulointerstitial kidney disease and hyperuricemia and gout. Functional studies clearly support the pathogenic role of the newly identified variant as mutant renin is retained in the ER, likely due to protein misfolding, it triggers ER stress and it affects pronephros development when expressed in zebrafish. This work extends the spectrum of *REN* mutations in ADTKD and suggests a broader phenotype in ADTKD-*REN* patients. Our findings have clear implications for the molecular diagnosis and the estimated prevalence of the disease and provide new insight on molecular pathogenesis.

## Results

### Clinical investigations

**Figure 1:**
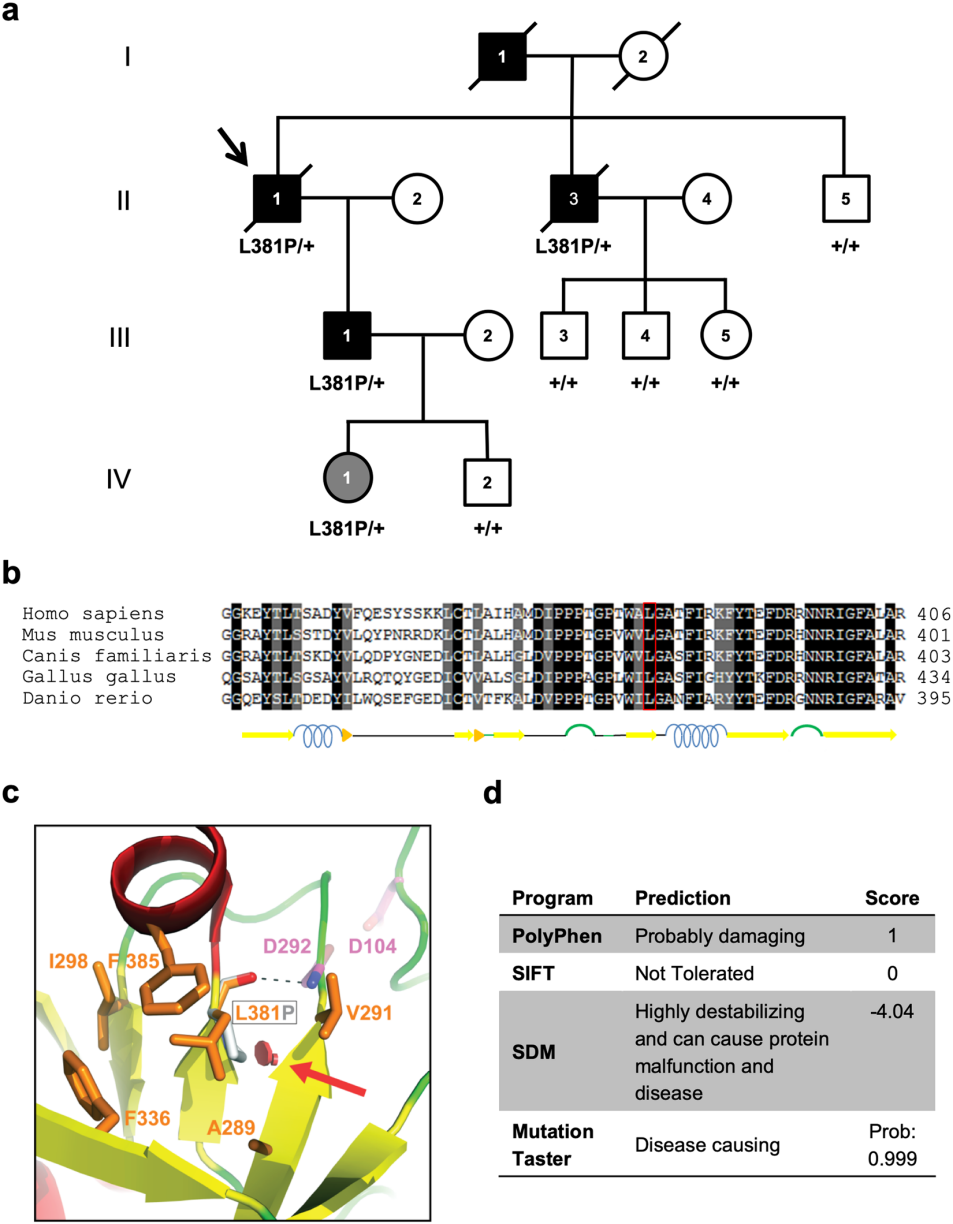
Identification of L381P renin variant in an ADTKD family. (**a**) Pedigree chart of the ADTKD family segregating the renin p.L381P variant. Index case (arrow): II-1. Black symbols denote clinically affected individuals, open symbols denote clinically unaffected individuals. Corresponding *REN* genotype is provided for each tested individual. (**b**) Alignment of renin sequence from human, mouse, dog, chicken and zebrafish performed with the Sequence Manipulation Suite^38^. Identical or similar residues are shown with a black and grey background respectively. Predicted secondary structure is shown below. (**c**) Detail of the human renin structure, shown in cartoon representation and colored by secondary structure (red: α-helices; yellow: β-strands; green: loops). L381 and nearby conserved hydrophobic residues (orange), as well as the two catalytic aspartates (magenta), are depicted as sticks; a red arrow indicates the steric clash (red discs) that would be introduced upon mutation of L381 to proline (grey stick). The main-chain hydrogen bond between L381 and D292 is represented by a dashed black line. The figure was made with PyMOL (Schrödinger LLC). (**d**) Predicted effect of the renin L381P variant, as assessed by using PolyPhen-2^29^, Sorting Intolerant From Tolerant (SIFT)^30^, Site Directed Mutator (SDM)^31^ and Mutation Taster^32^.

The index case (II-1, Fig. 1a) had a diagnosis of chronic tubulointerstitial nephritis and renal tubular acidosis type IV at age of 65; serum creatinine was 2.8 mg/dl; serum sodium 141 mEq/l; serum potassium 6.4 mEq/l, serum bicarbonate 20 mEq/l, serum uric acid 7.5 mg/dl. Morning serum cortisol level was normal. Plasma renin activity and serum aldosterone concentration were reduced, suggesting hyporeninemic hypoaldosteronism. Urinalysis was non-significant. Hypertension was present since the age of 40. Renal ultrasonography revealed small kidneys with few cysts. At age 71, he reached ESRD requiring dialysis. He died of myocardial infarction at age 83.

The father (I-1) of the index case died of uremia at age of 55. He was known to have suffered from renal disease for many years.

The brother of the index case (II-3) developed hypertension at age 42; tophaceous gout at age 45; chronic renal failure (serum creatinine 1.6 mg/dl) at age 50. At age 61, an abdominal ultrasound revealed two small kidneys; serum creatinine was 2.2 mg/dl; serum uric acid 8 mg/dl. Urinalysis was non-significant. At the age of 67, he developed ESRD requiring dialysis. The patient died at the age of 70 for colorectal cancer.

The youngest brother (II-5) of the index case was screened at age 70 for renal disease and proved to be negative.

The son (III-1) of the index case was found to have asymptomatic chronic kidney disease (serum creatinine 1.3 mg/dl, estimated Glomerular Filtration Rate [eGFR] 63.6 ml/min) when screened at age 50. Urinalysis was non-significant; urinary osmolarity 249 mOsm/kg. Renal ultrasound showed normal-sized kidneys with few cysts. Normal levels of serum electrolytes, serum cortisol, direct plasma renin concentration, plasma aldosterone concentration and 24-hour urinary aldosterone excretion were detected. Hemoglobin, reticulocyte count and erythropoietin levels were all in the normal range. The past medical history was unremarkable; blood pressure was 105/70 mmHg.

On the basis of the clinical data, a diagnosis of ADTKD was assigned to affected family members.

Other available family members II-2, II-4, III-2, III-3, III-4, III-5, IV-1 and IV-2 had no evidence of renal disease when screened in July 2017. A detailed clinical investigation was performed in the 19-year old subject IV-I harboring the L381P variant, which showed normal renal function (serum creatinine 0.8 mg/dl, eGFR 106 ml/min), negative urinalysis, normal renal ultrasound, normal blood count and erythropoietin levels. Uricemia was 3.5 mg/dl; plasma direct renin concentration, aldosterone concentration and 24-hour urinary aldosterone excretion were in the normal range. Her previous medical history was unremarkable. At the age of 10, laboratory tests, performed during an acute febrile episode, revealed normal renal function, negative urinalysis and a complete blood count within the normal range.

Clinical data of family members carrying renin p.L381P mutation are summarized in Table 1.

**Table 1:** Clinical data of family members carrying renin p.L381P mutation.

ID	Age at diagnosis	sCr mg/dl	HyperU/Gout	Renin-Angiotensin-Aldosterone System activity			K^+^ mEq/l	ESRD	Renal US
				Renin	Pl Ald pg/ml (37–310)	Ur Ald μg/24 h (2–25)			
II-1	61	2.8	Yes/no	PRA: 0.18 ng/ml/h (n.v. 0.2–3.4)	24	nd	6.4	Yes	Small kidneys, bilateral small cysts
II-3	50	2.2	Yes/yes	nd	nd	nd	5	Yes	Small kidneys
III-1	50	1.3	No/no	DRC:28 μIU/ml (n.v. 4.2–60)	81	18.5	3.7	—	Normal kidneys, bilateral small cysts
IV-1	19	0.81	No/no	DRC:27 μIU/ml	115	12.3	3.9	—	Normal kidneys

ID refers to pedigree in Fig. 1a; HyperU, hyperuricemia; Pl, plasma; Ur, urinary; Ald, aldosterone; PRA, plasma renin activity; DRC, direct renin concentration; ESRD, end-stage renal disease; US, ultrasound; nd, no data; n.v. normal values.

### Identification of a new variant in the *REN* gene

Mutations in the known ADTKD responsible genes *UMOD*, *HNF1B* and *REN* (exon 1) were excluded by sequencing analysis. With the aim to identify the gene responsible for the disease in this family, exome sequencing was performed on individuals II-1, II-3, II-5. We obtained an average exome coverage of 40X. From sequencing data we derived the allelic status and haplotype for each individual for 6 polymorphic loci in a region of about 130 kb centered on *MUC1*. The only *MUC1* haplotype shared by the two affected individuals was also present in the healthy brother, thereby strongly suggesting absence of involvement of the ADTKD gene *MUC1*. After quality control, we excluded variants already listed in polymorphism repositories (dbSNP) with minor allele frequency (MAF) greater than 0.01 or in Exome Variant Server with MAF greater than 0.001, or identified in 15 exomes from non-related, healthy Italian subjects. We finally identified 49 variants co-segregating with the disease (Supplementary Table S1). Interestingly, we identified a variant (c.1142T > C) in *REN* (exon 10) leading to the amino acid change L381P in the renin protein. This variant is not listed in any public repository of population-based exome sequencing project (ExAC, gnomAD) and was not found in 169 ethnically matched controls. The presence of the L381P variant was confirmed by Sanger sequencing in the two affected brothers (II-1 and II-3) and in one affected son (III-1) and it was excluded in the other brother (II-5) and three nephews of the proband (III-3, III-4 and III-5), all healthy, in accordance with a model of autosomal dominant inheritance (Fig. 1a). The youngest subjects of the investigated family were the daughter (IV-1, 19 years old) and the son (IV-2, 16 years old) of the affected subject III-1. While they were both healthy, individual IV-1 was found to be carrier of the L381P variant (Fig. 1a). Assuming a dominant model of inheritance with age-dependent penetrance (late-onset disease), hence excluding young subjects IV-1 and IV-2, the calculated probability for the observed variant-affected status data to occur by chance, rather than due to co-segregation, is N = 1/256 = 0.004, providing strong evidence of pathogenicity, according to the proposed classification of pathogenicity by the American College of Medical Genetics and the Association of Molecular Pathology^13^.

### Sequence conservation and pathogenicity prediction

In a first attempt to determine the potential pathogenicity of the L381P variant, we assessed the evolutionary conservation of Leucine 381. As shown by protein sequence alignment, this leucine residue is highly conserved, as it is found in virtually all renin homologues, from human to zebrafish (Fig. 1b). We also analyzed the possible consequences of the mutation at the structural level by taking advantage of available prorenin structure coordinates (PDB IDs 3VCM^14^ and 4AMT). The L381P substitution (corresponding to L301P in the pepsinogen-based prorenin numbering scheme^14^) affects a residue that contributes to the hydrophobic core of the protein, in proximity of invariant V291, F336, and F385 as well as conserved A289 and I298. Moreover, the carbonyl oxygen of L381 makes a hydrogen bond with the amide proton of D292, one of the two catalytic residues of renin (Fig. 1c). A change to proline is predicted to interfere with hydrophobic core packing and introduce a clash with the adjacent β-strand that immediately precedes D292. As a consequence, a significant effect on protein folding is expected. Consistently, when we evaluated the impact of the missense variant by using several *in silico* tools for pathogenicity prediction (PolyPhen-2, SIFT, SDM and Mutation Taster), all programs predicted that the identified variant L381P is deleterious, thus strongly suggesting a pathogenic effect (Fig. 1d).

### The L381P renin variant is retained in the ER and it is not secreted

**Figure 2:**
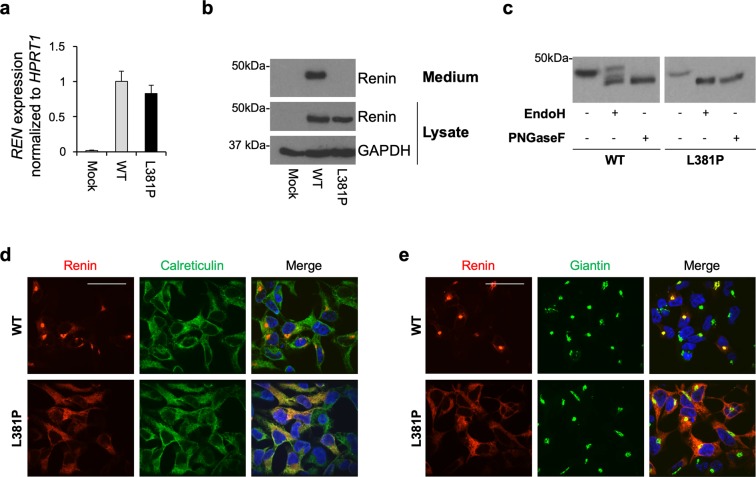
The L381P renin isoform is retained in the ER. (**a**) Renin expression in stably transfected HEK cells assessed by real-time RT-qPCR. Expression is normalized to *HPRT1*. Data are expressed as mean ± s.d. (n = 3 independent experiments). **(b)** Western blot analysis showing renin expression in cell lysate and conditioned medium. GAPDH is shown as a loading control. No secreted renin can be detected for the L381P isoform. **(c)** Western blot analysis showing renin in HEK lysates after treatment with EndoH or PNGaseF. Full-length blots (**b,c**) are presented in Supplementary Fig. S1. **(d)** Immunofluorescence analysis showing renin (red) and calreticulin (ER marker, green) and merged pictures with nuclei (dapi, blue). Bar = 40 μm. The variant L381P shows co-localization with calreticulin demonstrating its ER retention. **(e)** Immunofluorescence analysis showing renin (red) and giantin (Golgi marker, green) and merged pictures with nuclei (dapi, blue). Bar = 40 μm. Co-localization with giantin is seen for the wild type isoform only, demonstrating its trafficking along the secretory pathway.

To analyze the functional consequences of the missense change L381P on renin trafficking and secretion, we stably expressed HA-tagged wild type or L381P renin isoforms in HEK cells. The expression level of renin in both cell lines is comparable, as determined by quantitative real-time RT-PCR (Fig. 2a). Western blot analysis of renin in cell lysates and conditioned media shows total absence of secretion of the L381P isoform (Fig. 2b). Deglycosylation of the cell lysates by EndoH, specific for high-mannose, ER-type N-glycans, and PNGaseF, digesting all N-glycans, shows that the L381P variant is fully EndoH sensitive, demonstrating that this variant is uniquely present as a high-mannose ER precursor, while the wild type isoform carries complex, Golgi-type glycosylation (Fig. 2c). ER retention of the L381P variant was also confirmed by immunofluorescence analysis showing its co-localization with the ER marker calreticulin (Fig. 2d), as opposed to the wild type isoform that enters the secretory pathway and mainly co-localized with the Golgi marker giantin (Fig. 2e). Altogether these results demonstrate that the L381P variant is fully retained in the ER and does not proceed along the secretory pathway.

### The L381P renin variant induces ER stress and the Unfolded Protein Response

**Figure 3:**
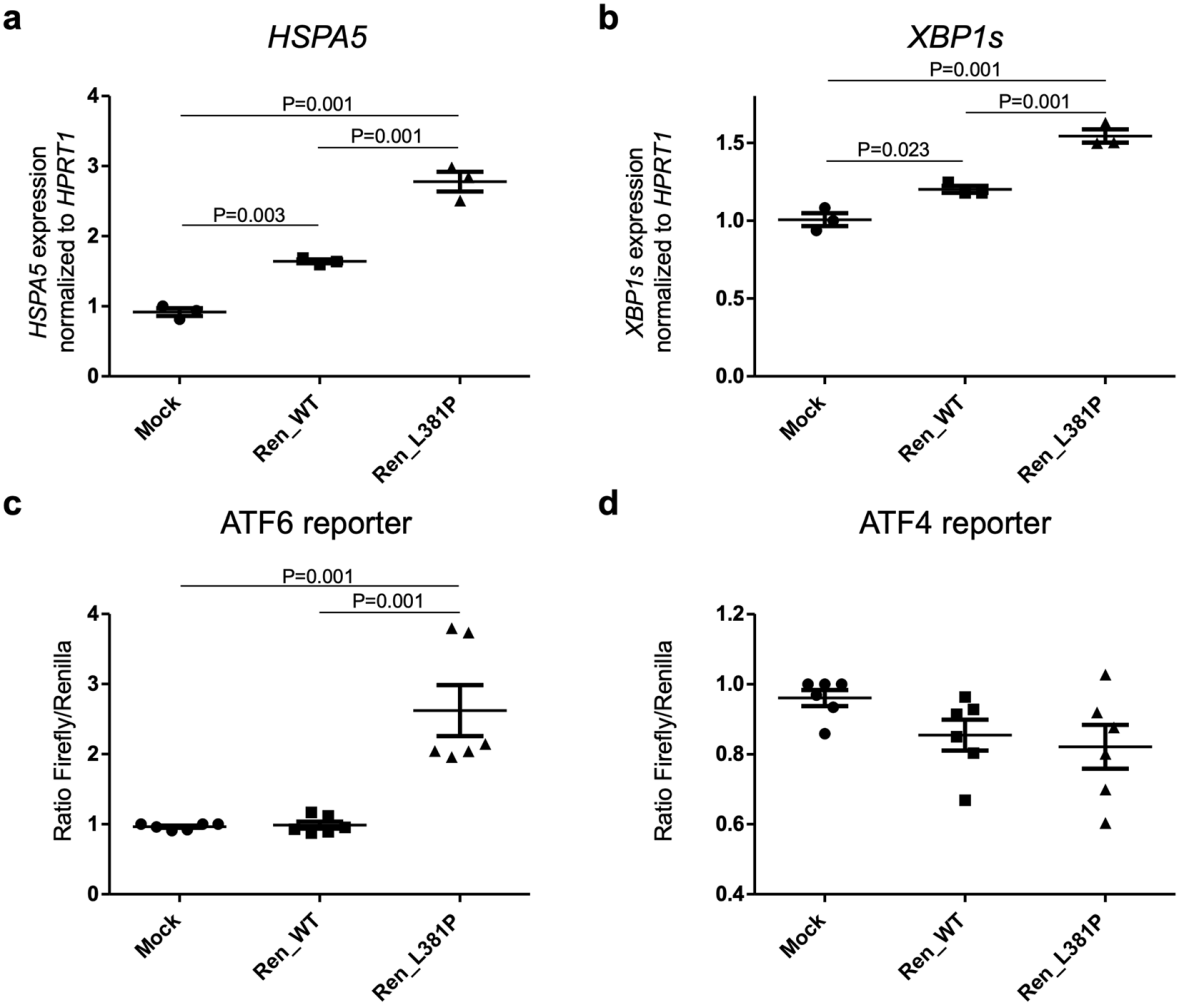
Expression of L381P renin isoform induces the UPR. **(a)** *HSPA5* and (**b**) *XBP1s* expression analysis by real-time RT-qPCR. Gene expression is normalized to *HPRT1*. Expression of L381P isoform in HEK cells induces expression of *HSPA5* (BiP) and spliced *XBP1*, markers of ER stress and UPR respectively. Data are expressed as mean ± s.d. (n = 3 independent experiments) (one way ANOVA, P = 1.78 ×    10^−5^ and P = 1 × 10^−4^ for *HSPA5* and spliced *XBP1* respectively, followed by Tukey Honest Significant Difference (HSD) *post hoc* test). **(c)** ATF6 activation assessed through the use of a luciferase-based, ATF6 reporter construct. ATF6 activation is observed upon expression of the L381P renin isoform. Data are expressed as mean ± s.d. (n = 6 independent experiments) (one way ANOVA, P = 5.78 × 10^−5^, followed by Tukey HSD *post hoc* test). **(d)** ATF4 activity, assessed through the use of a luciferase-based reporter construct. No increase of luciferase activity is observed in cells expressing the L381P renin isoform compared to mock or cells expressing wild type renin. Data are expressed as mean ± s.d. (n = 6 independent experiments) (one way ANOVA, P = 0.1175).

Previous functional study on L16del renin mutant, a dominant renin mutant isoform responsible for ADTKD, showed that its expression induces the UPR, a cellular response to perturbed ER homeostasis^4^. In particular, renin L16del was shown to induce splicing of XBP1 (XBP1s), the main target of IRE1, one of the three UPR cellular sensors. To assess if ER retention of the L381P renin variant induces ER stress, we analyzed the expression level of *HSPA5* encoding the main ER chaperone Bip (Immunoglobulin heavy chain-binding protein). RT-qPCR showed increased expression of this chaperone in mutant -expressing cells compared to cells expressing wild type renin and mock control cells (Fig. 3a). Expression of renin L381P also induces splicing of Xbp1 (Fig. 3b) and it also activates the ATF6 pathway of the UPR, as shown by increased activation of a well-established ATF6 reporter construct (Fig. 3c)^15^. We did not observe induction of the PERK branch of the UPR, as revealed by using a luciferase-based reporter construct (Fig. 3d)^16^. Hence, the L381P renin isoform induces ER stress and the UPR, further supporting its pathogenic role.

### The L381P renin variant does not interfere with wild type renin trafficking

**Figure 4:**
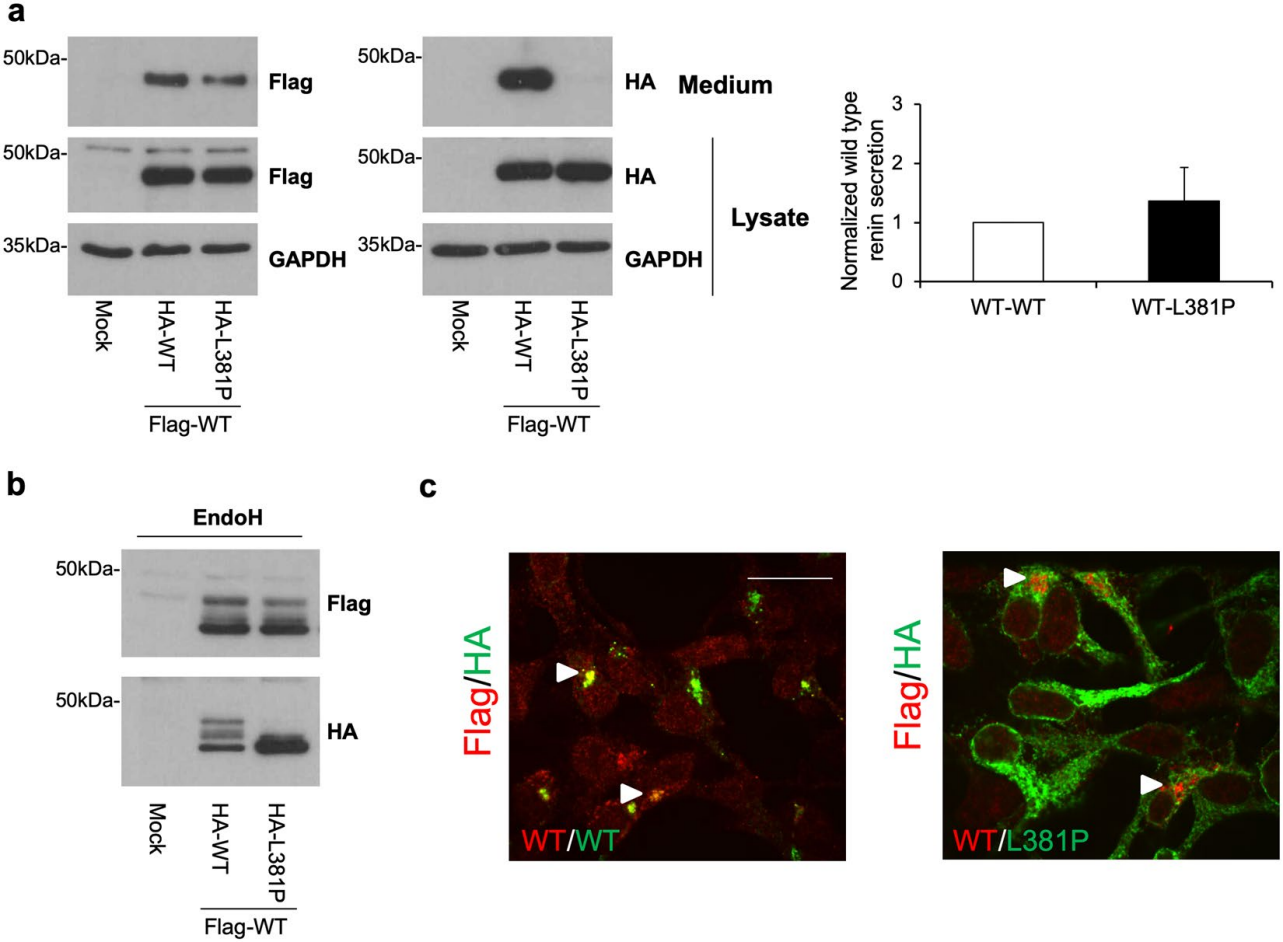
The L381P renin isoform does not exert a dominant negative effect on trafficking of wild type renin. **(a)** Western blot analysis showing Flag- and HA-tagged renin isoforms in lysate and conditioned medium of stably transfected HEK cells. GAPDH is shown as a loading control. Quantification of secreted Flag-tagged wild type isoform normalized to Flag-tagged wild type protein present in cell lysate is shown. Data are expressed as mean ± s.d. (n = 3 independent experiments). **(b)** Western blot analysis showing Flag- and HA-tagged renin in HEK lysates after treatment with EndoH. Full-length blots (**a,b**) are presented in Supplementary Fig. S3. **(c)** Immunofluorescence analysis showing merged picture of Flag- and HA-tagged renin in red and green respectively. Bar = 40 μm. Cellular localization of wild type renin is not affected by co-expression with ER-retained L381P isoform.

To mimic the patient heterozygous state, wild type and L381P mutant renin were stably co-expressed in HEK cells. Equal expression level of the two isoforms was ensured by using a bicistronic expression vector. Wild type renin (Flag-tagged) was co-expressed with either wild type or L381P isoforms (HA-tagged) (Supplementary Fig. S2). Co-expression of L381P renin did not affect secretion of the wild type protein (Fig. 4a). Moreover, the band pattern observed for each isoform at baseline or after deglycosylation with EndoH was similar when expressed alone or in combination, suggesting absence of any dominant negative effect (Fig. 4b). This is further supported by immunofluorescence experiments showing no co-localization of wild type and L381P renin isoforms. Indeed, while wild type renin is mainly localized in the Golgi, the L381P isoform is retained in the ER (Fig. 4c). Taken together these data demonstrate that despite ER retention of mutant protein, trafficking of co-expressed wild type renin is not altered.

### L381P renin induces alteration of the pronephros in zebrafish embryos

**Figure 5:**
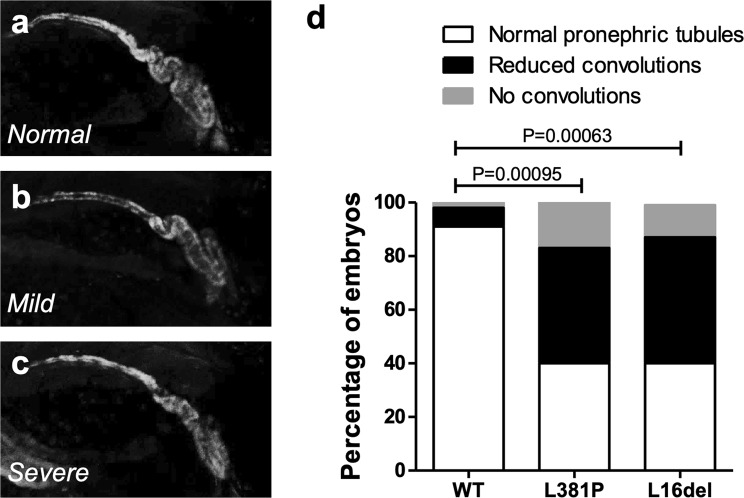
Expression of L381P renin variant induces alteration in the pronephros of zebrafish embryos. The pronephros of 5 dpf zebrafish embryos is labelled by reporter transgene EGFP (line ia20Tg^17^). (**a–c)** Embryos injected with wild type (WT), L381P and L16del renin mRNAs show three different phenotypes: normal convoluted pronephric tubules; tubules with reduced convolutions (mild phenotype); absence of tubular convolution (severe phenotype). **(d)** Histograms showing the percentage of embryos with normal pronephros, or mild or severe pronephric alterations. Samples injected with renin mutants L381P (n = 112) and L16del (n = 122) show significantly increased frequency of altered pronephric tubules relative to wild type (n = 183) (chi-square analysis).

To analyze *in vivo* the putative pathogenic role of the renin variant identified in ADTKD patients, we evaluated the effects induced by expression of human L381P renin mRNA in zebrafish (*Danio rerio*) embryos. The zebrafish genome encodes a single *renin* orthologue (GenBank NP_998025.1), with 70% similarity and 53% identity at amino acidic level to human renin. One-cell stage zebrafish embryos were injected with either wild type or L381P renin mRNAs. As reference, we also assessed in zebrafish the effects induced by the overexpression of the well-established, ADTKD related, renin mutant L16del^4^. Injected embryos had a normal development without showing overt phenotypical alterations or growth retardation. At 5 days post fertilization (dpf) no significant alterations in the head, heart, and swim bladder were detected. In zebrafish, renin is expressed in the region of the developing pronephros starting from 1.5 dpf. We tested if renin overexpression affects pronephros development by using a recently developed transgenic reporter line for glucocorticoid transcriptional activity (ia20Tg), already shown to have robust expression of EGFP in the pronephros at 2–6 dpf^17^. Embryos were scored in three phenotypic classes according to pronephros organization: (i) normal convoluted pronephric tubules; (ii) tubules with reduced convolution; (iii) absence of tubular convolution (Fig. 5a–c). Interestingly, 60% of embryos injected with the L381P renin variant showed pronephric alteration: 43% were characterized by reduced, partial convolution of the pronephric tubules and 17% showed absence of tubular convolutions (Fig. 5d). A similar phenotypic distribution was observed in embryos injected with the L16del mutation (40% normal; 48% mild phenotype; 12% severe phenotype). By contrast, only 9% of embryos injected with wild type renin showed alterations of pronephric tubules (Fig. 5d). Taken together, this *in vivo* functional analysis suggests that overexpression of the L381P renin variant exerts a pathogenic effect altering the correct organization of the pronephros in zebrafish embryos.

## Discussion

In this study we identified a new dominant renin mutation (p.L381P) in an ADTKD pedigree. Interestingly, it is the first time that a mutation responsible for ADTKD-*REN* is found outside of the renin leader peptide. According to the guidelines for pathogenicity prediction of DNA variants of the American College of Medical Genetics and of the Association of Molecular Pathology^18^, the p.L381P (c.1142T > C) variant is likely pathogenic, since it meets three strong criteria as co-segregation, computational and predictive data, and functional evidence. First, this variant is not found in control individuals, including public repositories of genomic data and 169 ethnically matched controls. Second, it affects a residue in mature renin that is highly conserved during evolution. Third, we demonstrate that the L381P substitution leads to mutant protein ER retention and activation of ER stress pathways. Its pathogenicity is strongly supported by the fact that its expression in zebrafish embryo leads to convolution defect during the development of the pronephros.

To date, only 6 families with established ADTKD-*REN* have been reported in the literature^4–7^. They all harbor dominant mutations mapping in the first exon of the gene, affecting residues within the protein leader peptide and lead to very early onset of clinical manifestations. In these families, impaired kidney function can be found as early as three or four years of age^4,6^. CKD shows a slowly progressive course, and affected subjects reach ESRD in the fifth or sixth decade of life. The hypo-proliferative anemia also begins early in life, since it has been found even in one/three-year-old affected children. Interestingly, anemia resolves as the child enters adolescence, as long as renal function is not severely impaired. Finally, hyperuricemia and gout, usually develop in the second decade of life. However, due to the small number of ADTKD-*REN* described families, the spectrum of clinical manifestations may not yet be fully appreciated. Although the renal phenotype in affected family members carrying the L381P mutation is in line with this “classical” clinical picture, some relevant differences were observed. Indeed, in our family the new mutation p.L381P was associated with milder renal phenotype, mainly characterized by late onset of kidney disease and of hyperuricemia and gout. The first signs of renal dysfunction appeared in adulthood, at 50–60 years of age. End-stage renal disease was reached approximately at the age of 70. The son of the index case was found to have mild, asymptomatic, renal disease (CKD stage 2) at age 50, and the youngest carrier of the p.L381P variant (subject IV-1) did not show signs of renal disease at age 19. Adult onset was also observed for gout, diagnosed in one affected family member (II-3) at age 45. With respect to the hypo-proliferative anemia, it was not detected in the youngest mutated subject (IV-1) when studied at age 10. We were unable to test for young-onset anemia in the affected patients, since they came to our attention in adult age.

Notably, although the newly identified L381P mutation maps in a different region compared to the previously described ones, they share some common molecular pathways. This new mutation likely affects protein folding, as shown by structural prediction analysis and by the fact that, contrary to the wild type isoform, the mutant protein is trapped inside the ER and is not secreted. ER retention of the mutant protein induces UPR as already described for L16del mutation^4^, while it does not exert a dominant negative effect on wild type protein trafficking. To better understand the potential pathogenic role of L381P variant we carried out the first study of dominant, ADTKD-related *REN* mutations *in vivo* in a zebrafish model. Interestingly, expression of mutant renin was associated with convolution defect of the developing pronephros. We observed such effect also for L16del mutation, suggesting a common, gain-of-function mechanism of dominant renin mutations that is consistent with genetic data. Indeed, lack of one renin functional allele, as in heterozygous carriers of *REN* recessive mutations associated with RTD, is not sufficient to induce a renal phenotype. Building on our data and previously published evidence, ER stress and activation of the UPR appear as the common end-point for *REN* mutations despite their different cellular phenotype (defective insertion in the ER for L16del^4^ and accumulation inside the ER for L381P). Differences in the clinical manifestation of the disease could be explained by quantitative and qualitative differences in cell stress pathway activation. We speculate that, in addition to UPR, mutants in the leader peptide could also induce cytosolic stress pathways, as already described for mutations in the leader peptide of preproinsulin associated with maturity onset diabetes of the young (MODY)^19^. In view of the results emerging from ADTKD literature, it is interesting to note that ER homeostasis could actually be a common denominator not only for ADTKD-*REN* but more generally for all forms part of this heterogeneous disease. First, in the case of *UMOD*, the ADTKD gene for which mutations have been studied the most at the molecular level, ER stress and induction of pro-inflammatory cytokines have been identified as important factors in the disease pathogenesis^20–25^. Second, ADTKD-*MUC1* is likely due to altered protein homeostasis as intracellular accumulation of aberrant, frameshifted protein MUC1-fs is necessary to trigger kidney disease^1^. Third, the discovery of ADTKD mutations in *SEC61A1*, one of the main constituents of the ER translocon, reinforces the idea that perturbation of ER homeostasis could lead to the establishment of tubulo-interstitial fibrosis^26^. Along this line it is interesting to note that a pronephros convolution defect similar to the one here described for *REN* mutants, was reported in zebrafish upon suppression of *sec61a1*^26^, modelling *SEC61A1* loss-of-function mutations.

Kidney fibrosis, one of the hallmarks of ADTKD, can indeed be induced by ER stress and UPR^27^. The molecular mechanisms are not fully understood, however different pathways could be evoked, as apoptosis of epithelial cells, induction of pro-fibrotic cytokine expression and activation of myofibroblasts^28^. To date, there is no specific therapy proposed for ADTKD-*REN* except for fludrocortisone that has been successfully used in one patient^5^. As fludrocortisone should decrease renin production (including mutant isoform) via feedback inhibition, it was hypothesized that it should be beneficial to preserve renal function. Along the same line of thinking, we believe that renal disease caused by the L381P mutant could be alleviated by using chemical chaperones that may rescue mutant renin trafficking along the secretory pathway.

In conclusion, our work shows that additional *REN* mutations, other than the ones mapping in exon 1 of the gene and affecting the protein leader peptide, can cause ADTKD. This has implications for the molecular diagnosis and the estimated prevalence of the disease. In addition, our study delineates an adult form of ADTKD-*REN*, a condition which has its onset in childhood or adolescence, usually requiring a pediatric nephrology referral^7^. Finally, our study points at ER homeostasis as a common pathway affected in ADTKD-*REN*, and possibly more generally in ADTKD, that may represent a promising therapeutic target.

## Methods

### Patients and diagnostic criteria

A family of Italian ancestry (Fig. 1a) was ascertained at the Section on Nephrology, University School of Medicine of Brescia, Italy. Medical histories were obtained as part of all patients’ clinical work-up. Clinical and genetic data were collected according to national laws. Observational clinical studies (case report or case series report) do not require approval by the ethics committee according to AIFA (Italian Medicines Agency) statement 20-03-2008 published in G.U. n. 76 of 31-3-2008. All patients signed the informed consent for genetic testing which include the authorization of the processing of clinical and genetic data for research purposes in compliance with local (circ.reg 28.05.2013) and national legislations (General Authorization n°8 of Garante Privacy) and according to General Data Protection Regulation (Regulation (EU) 2016/679 of the EU Parliament and of the Council of 27 April 2016) and to the Helsinki Declaration. Family tree was obtained by personal interview of the patients and their relatives. Diagnosis of ADTKD was established on the basis of clinical features compatible with chronic tubulointerstitial kidney disease (renal impairment and bland urinalysis, hyperuricemia and gout), family history with dominant mode of inheritance. At-risk adult family members were offered a pre-symptomatic genetic testing. Carriers of the mutation underwent a clinical, laboratory, and instrumental assessment, including renal function tests and abdominal ultrasonography. DNA was obtained using standard procedures from peripheral blood mononuclear cells of affected and healthy at-risk family members. The 169 ethnically matched control subjects are Italian patients from the same geographical area (Brescia) as the ADTKD family here reported. These subjects underwent targeted gene panel sequencing, including *PKD2*, *UMOD*, *HNF1B*, *REN*, *TSC1* and *TSC2*, and were diagnosed with polycystic kidney disease (identified mutation in either *PKD1* or *PKD2*). The average coverage of *REN* gene exon 10 (where the c.1142T > C, p.L381P variant maps) was about 1000x.

### Constructs

#### Human renin constructs

Human renin cDNA was cloned in pcDNA3.1 (Thermofisher, Waltham, MA) or in pVITRO-hygro-mcs (Invivogen, San Diego, CA) for co-expression experiments. Indicated tags were inserted at the protein C-terminal. Further details are reported in Supplementary Methods.

#### Luciferase reporter constructs

The reporter construct for ATF6, p5xATF6-GL3, was created by Prof. Ron Prywes^15^ and was obtained from Addgene (Watertown, MA) (Addgene plasmid # 11976). The reporter construct for ATF4 (ATF4 3: Mouse ATF4 (CHOP11/cATF), 5′UTR and AUG-luc) was created by Prof. David Ron^16^ and obtained from Addgene (Addgene plasmid # 21850).

### Cell line

HEK cells were grown in DMEM (Thermofisher) supplemented with 10% fetal bovine serum (Euroclone, Pero, Italy), 200 U/mL penicillin, 200 µg/mL streptomycin and 2 mM glutamine (Thermofisher) at 37 °C, 5% CO_2_. Stable populations were generated by transfecting HEK cells with Lipofectamine 2000 (Thermofisher) following the manufacturer’s protocol. See Supplementary Methods for further details.

### Real time quantitative RT-PCR

RNA extraction and PCR were performed as described in Schaeffer *et al*.^20^.  Primers for real time RT-qPCR are reported in Supplementary Methods.

### Western blot

Protein lysates, deglycosylation experiments and western blot experiments were performed as described in Schaeffer *et al*.^20^. Detailed protocol and list of used antibodies is available in Supplementary Methods.

### Immunofluorescence

Immunofluorescence experiments were performed as described in Schaeffer *et al.*^20^. The list of used antibodies is available in Supplementary Methods.

### Luciferase assay

HEK cells stably expressing the indicated renin isoform were plated in 12 well plate and transfected with 490 ng or 990 ng of ATF4 or ATF6 reporter constructs respectively together with 10 ng of pGL4.73[hRluc/SV40] (Promega, Madison, WI) using lipofectamine 2000 (Thermofisher). Firefly and Renilla luciferase were measured 36 hours after transfection using the Dual-Luciferase® Reporter Assay System (Promega).

### Exome sequencing

TruSeqTM Exome Enrichment Preparation Kit (Illumina, San Diego, CA) was used for exome DNA library preparation following manufacturer’s instructions. Each library was run on the HiSeq 2500 (Illumina), performing pair-end sequencing (2 × 101) based on the sequencing by synthesis (SBS) protocol. Further details are provided in Supplementary Methods.

### *In silico* studies

Pathogenicity of the p.L381P renin variant was predicted using the following *in silico* tools: PolyPhen-2^29^, Sorting Intolerant From Tolerant (SIFT)^30^, Site Directed Mutator (SDM)^31^ and Mutation Taster^32^. The effect of the L381P mutation was predicted with the standalone version of FoldX^33^ using as starting point the re-refined coordinates of PDB entry 4AMT in the PDB-REDO databank^34^. This model was prepared using the RepairPDB command of FoldX and subsequently subjected to 5 BuildModel runs, resulting in an average free energy difference between the wild-type and the mutant of 3.1 Kcal/mol (consistent with a destabilizing mutation).

### *In vivo* functional study of L381P renin variant

Zebrafish embryos and adults were maintained and mated according to standard procedures^35^. Using a standard protocol^36^, wild type, L381P and L16del renin mRNA were synthesized with the SP6 mMESSAGE mMACHINE kit (Ambion, ThermoFisher) using as template the pCS2_RenHA_WT, pCS2_RenHA_L381P and pCS2_RenHA_L16del plasmids. As previously described^37^, one-cell stage zebrafish embryos were injected with 200 pg of REN-WT, REN-L381P or REN-L16del mRNA and then fixed at 5 dpf. Injections of mutant and wild type renin mRNA were repeated in three independent experiments and a chi-square test was used to determine significance. Differences were considered significant for P < 0.05. All experiments were carried in accordance to Italian law on animal experimentation (D.L. 4 March 2014, n.26), under authorization n. 407/2015-PR from the Italian Ministry of Health.

## Supplementary information

Supplementary Information
