# Supplementary material for: Autosomal Dominant Tubulointerstitial Kidney Disease with Adult Onset due to a Novel Renin Mutation Mapping in the Mature Protein
Source: Sci Rep. 2019 Aug 12;9:11601. doi: 10.1038/s41598-019-48014-6 (PMC6691008; doi:10.1038/s41598-019-48014-6)
Supplement: Supplementary file 1 — Supplementary Information [file 41598_2019_48014_MOESM1_ESM.pdf]

Autosomal Dominant Tubulointerstitial Kidney Disease with Adult Onset due to a Novel Renin Mutation Mapping in the Mature Protein

Céline Schaeffer, Claudia Izzi, Andrea Vettori, Elena Pasqualetto, Davide Cittaro, Dejan Lazarevic, Gianluca Caridi, Barbara Gnutti, Cinzia Mazza, Luca Jovine, Francesco Scolari, Luca Rampoldi

Supplementary information

-Supplementary Methods
-Supplementary Figure S1
-Supplementary Figure S2
-Supplementary Figure S3
-Supplementary Table S1

SUPPLEMENTARY METHODS

Constructs

Human renin constructs

Human renin cDNA untagged clone (SC321832) was purchased from Origene (Rockville, MD) and subcloned in pcDNA3.1 (Thermofisher, Waltham, MA). HA or Flag tag were inserted at the protein C-terminus through 2 rounds of mutagenesis using the QuikChange Lightning Site-Directed Mutagenesis Kit (Agilent, Santa Clara, CA) following the manufacturer's instructions. The L381P mutation was introduced in the pcDNA-hRenHA construct using the QuikChange Lightning Site-Directed Mutagenesis Kit (Agilent). Mutagenesis primers were designed using the software QuikChange® Primer Design Program.

**Tag/Mutation**	**Primer Forward (5'>3')**	**Primer Reverse (5'>3')**
HA_1_Ren	GCCTTGGCCCGCTACCCATAC GATTGAGGCCCTCTG	CAGAGGGCCTCAATCGTAT GGGTAGCGGGCCAAGGCC
HA_2_Ren	GCCCGCTACCCATACGATGTT CCAGATTACGCTTGAGGCCCT	GAGGGCCTCAAGCGTAATC TGGAACATCGTATGGGTAG
Flag_1_Ren	GCCTTGGCCCGCGACTACAAG GATTGAGGCCCTCTG	CAGAGGGCCTCAATCCTTGT AGTCGCGGGCCAAGGC
Flag_2_Ren	GCCCGCGACTACAAGGATGAC GATGACAAATGAGGCCCTC	GAGGGCCTCATTTGTCATCG TCATCCTTGTAGTCGCGGGC
hREN L381P	AGGTGGCCCCCGGGGCCCAGG TG	CACCTGGGCCCCGGGGGCC ACCT

For co-expression experiments, Flag-tagged wild-type and HA-tagged renin isoforms were subcloned in the dual expression plasmid pVITRO-hygro-mcs (Invivogen, San Diego, CA). For experiments performed in zebrafish, HA-tagged constructs were subcloned in the pCS2+ vector.

Generation of HEK cell lines stably expressing human renin

Stable populations were generated by transfecting HEK cells with Lipofectamine 2000 (Thermofisher) following the manufacturer's protocol. For pcDNA and pVITRO constructs, selection was started 24 h after transfection by adding 500 µg/ml G418 (Invitrogen) or 200

µg/ml hygromycin (Thermofisher) respectively, and was pursued for 2 weeks to obtain a population of resistant cells.

Real-time quantitative RT-PCR

Quantitative real-time RT-PCR was performed on the LightCycler 480 instrument (Roche, Basel, Switzerland) using the qPCR Core kit for SYBR® Green I No ROX (Eurogentec, Liège, Belgium) with the gene-specific primers indicated below.

**Target gene**	**Primer Forward (5'>3')**	**Primer Reverse (5'>3')**
Ren (Human)	GGGCTCCTGTACCTTTGGTC	TCTTCATGGGTTGGCTCCAC
HSPA5	CGCTGAGGCTTATTTGGGAAA	TGCCGTAGGCTCGTTGATG
XBP1S	GAGTCCGCAGCAGGTG	ATACCGCCAGAATCCATGG
HPRT1	AGCCCTGGCGTCGTGATTAGT	TGTGATGGCCTCCCATCTCC

Western blot

HEK cells were grown in 35 mm dishes in complete medium. Cells were lysed in 300 µl of octylglucoside lysis buffer (50 mM Tris-HCl, pH 7.4, 150 mM NaCl, 60 mM octyl β-D-glucopyranoside, 10 mM NaF, 0.5 mM Sodium orthovanadate, 1 mM glycerophosphate and protease inhibitor cocktail (Sigma-Aldrich, St Louis, MO) for 1 h at 4°C under rotation followed by centrifugation 10 min at 17 000g. Soluble fractions were quantified by the Bio-Rad Protein Assay (Bio-Rad, Hercules, CA). When indicated, protein lysates were deglycosylated with peptide-N-glycosidase F (PNGase F) or endoglycosidase H (Endo H) (New England Biolabs, Ipswich, MA). Twenty to fifty µg of each protein lysate were loaded onto reducing SDS-polyacrylamide gel electrophoresis (PAGE). Transblotted nitrocellulose membranes (GE Healthcare, Little Chalfont, United Kingdom) were incubated with the indicated primary antibody followed by incubation with horseradish peroxidase-conjugated secondary antibody (1:7500 dilution; GE Healthcare). Protein bands were visualized with the Immobilon Western Chemiluminescent Horseradish Peroxidase Substrate kit (Millipore, Billerica, MA). Quantification was performed using ImageJ software [1].

Antibodies

Western blot

Mouse purified anti-HA.11 Epitope Tag antibody (dilution 1:1,000; Cat# 901502, Biolegend, San Diego, CA), mouse monoclonal anti-GAPDH antibody (dilution 1:1,000; sc-32233,

Santa Cruz Biotechnology, Dallas, TX) and rabbit affinity isolated anti-Flag antibody (dilution 1:1,000; F7425, Sigma-Aldrich).

Immunofluorescence

Mouse purified anti-HA.11 Epitope Tag antibody (dilution 1:500; Cat# 901502, Biolegend), rabbit affinity isolated anti-Flag antibody (dilution 1:500; F7425, Sigma-Aldrich), rabbit polyclonal anti-giantin antibody (dilution 1:500; Cat#PRB-114C, Covance, Princeton, NJ) and rabbit anti-calreticulin antibody (dilution 1:500; C4606, Sigma-Aldrich).

Exome sequencing

1μg of gDNA extracted from whole blood was sheered using E220 Focused-ultrasonicators (Covaris, Matthews, NC) under standard condition (vol.: 52 μl; treatment time: 120.0s; Duty Factor: 10 %; PiP 175 W; Cycle per brusts: 200). TruSeqTM Exome Enrichment Preparation Kit (Illumina, San Diego, CA) was used for exome DNA library preparation following manufacturer's instructions. Libraries from each patient were labelled with different barcodes allowing multiplex sample loading onto one lane. Before sequencing, each library was run on HS DNA Chip (Agilent) to assess quality. Each library was run on the HiSeq 2500 (Illumina), performing pair-end sequencing (2x101) based on SBS protocol. Reads were aligned to reference genome hg19 using bwa (v 0.6.1) [2], duplicated reads were marked using Picard MarkDuplicates. In order to call SNP and Indels, GATK UnifiedGenotyper (v.2.2.8) [3] was used following GATK best practices [4]. The resulting VCF file was annotated for impact using snpEff (v 1.8) [5] and matched against dbSNP v137. Non-synonymous variants affecting coding sequence and annotated with a MAF lower than 0.01 were retained for further analysis.

REFERENCES

1 Schneider, C. A., Rasband, W. S. & Eliceiri, K. W. NIH Image to ImageJ: 25 years of image analysis. *Nat Methods* **9**, 671-675 doi: 10.1038/nmeth.2089 (2012).

2 Li, H. & Durbin, R. Fast and accurate long-read alignment with Burrows-Wheeler transform. *Bioinformatics* **26**, 589-595, doi:10.1093/bioinformatics/btp698 (2010).

3 McKenna, A. *et al.* The Genome Analysis Toolkit: a MapReduce framework for analyzing next-generation DNA sequencing data. *Genome Res* **20**, 1297-1303, doi:10.1101/gr.107524.110 (2010).

4 DePristo, M. A. *et al.* A framework for variation discovery and genotyping using next-generation DNA sequencing data. *Nat Genet* **43**, 491-498, doi:10.1038/ng.806 (2011).

5 Cingolani, P. *et al.* A program for annotating and predicting the effects of single nucleotide polymorphisms, SnpEff: SNPs in the genome of Drosophila melanogaster strain w1118; iso-2; iso-3. *Fly (Austin)* **6**, 80-92, doi:10.4161/fly.19695 (2012).

Panel b

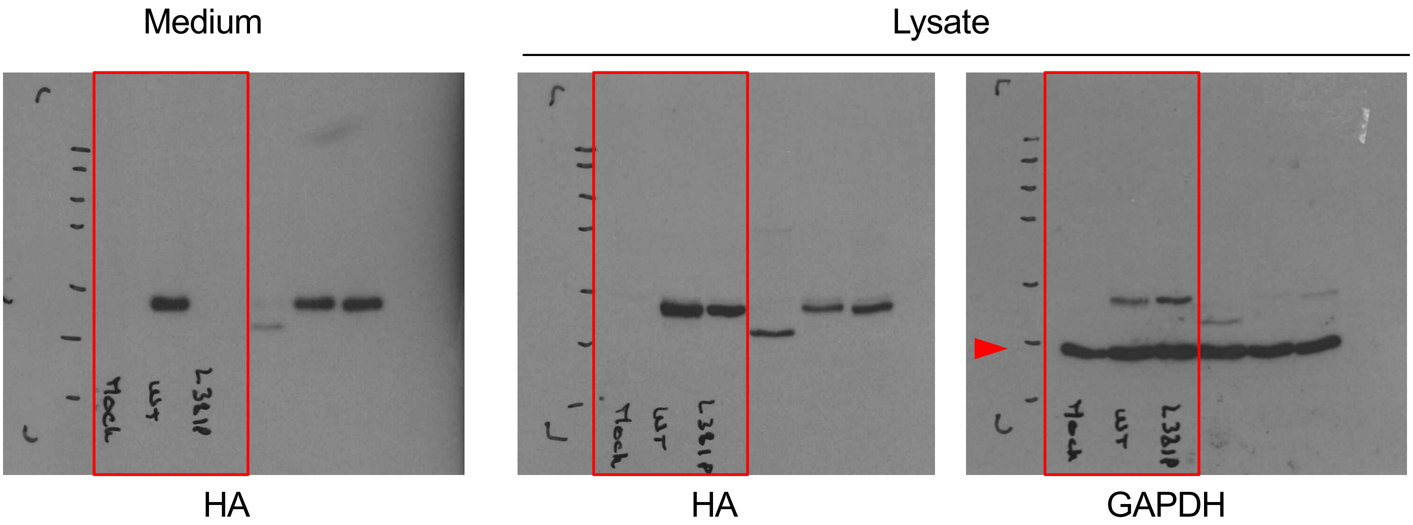

Panel c

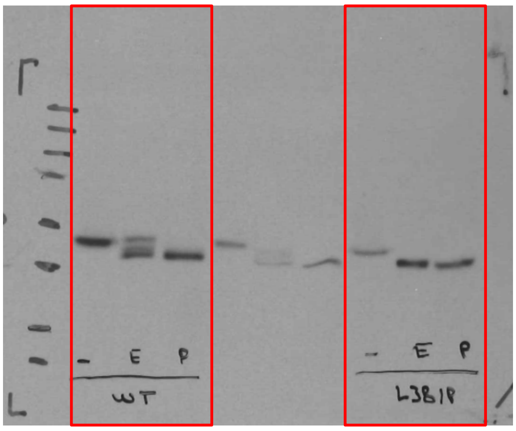

Supplementary Figure S1. Full-length blots, shown as cropped images in Figure 2b and c.
.

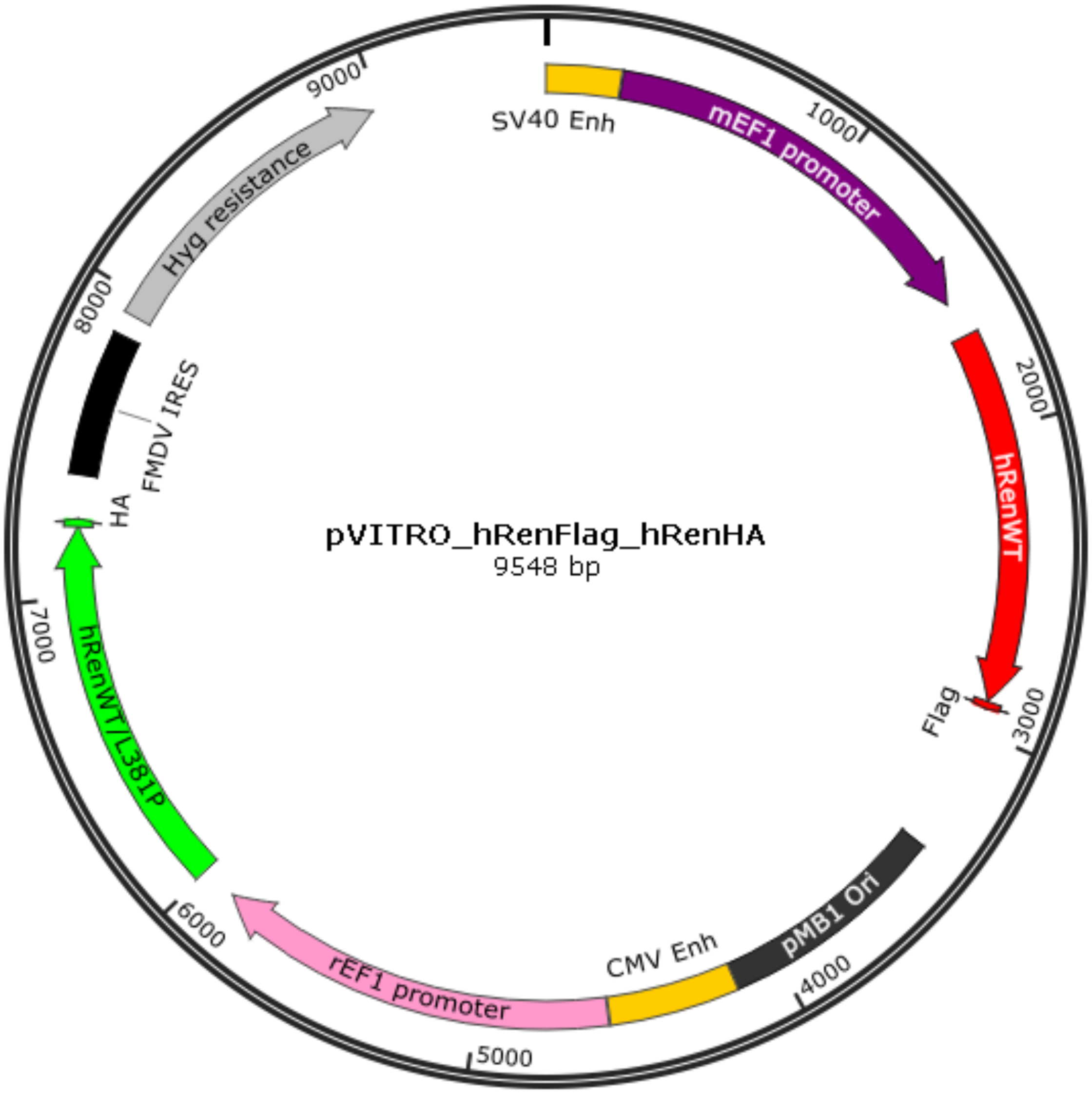

Supplementary Figure S2. Map of the plasmid used for co-expression of wild type renin (Flag-tagged) (red) with either wild type or L381P isoforms (HA-tagged) (green). The mouse Elongation Factor 1 (EF1) promoter (mEF1, purple) and the rat EF1 promoter (rEF1, pink) are used to drive comparable expression levels of co-expressed isoforms. The following, additional elements are indicated: Simian Virus 40 and Cytomegalovirus enhancers (SV40 Enh and CMV Enh) (yellow); minimal *E. coli* origin of replication (pMB1 Ori) (dark grey); internal ribosome entry site of the Foot and Mouth Disease Virus (FMDV IRES) (black); *hph* gene that confers resistance to Hygromycin B (Hyg resistance) (light grey).

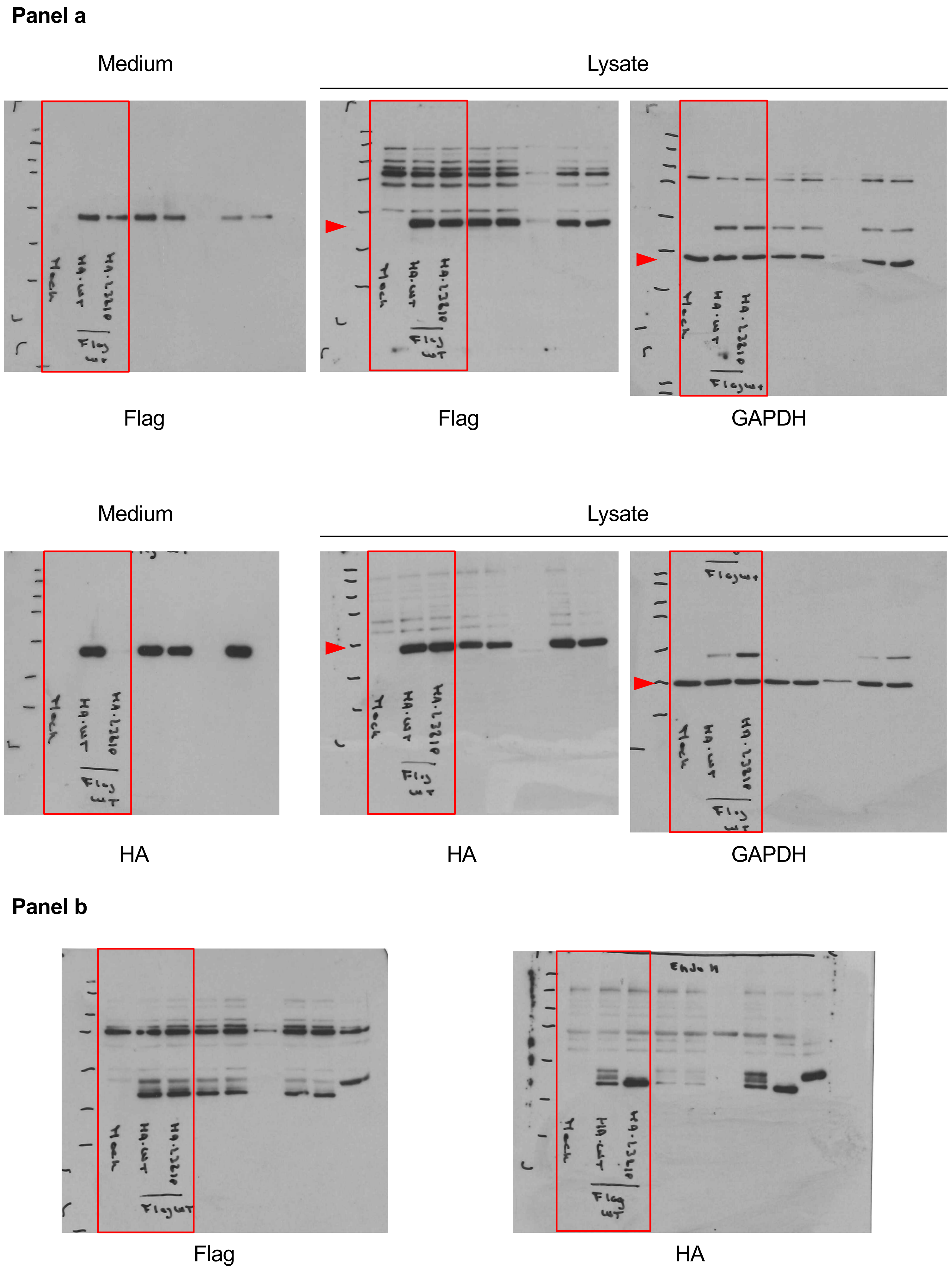

Supplementary Figure S3. Full-length blots, shown as cropped images in Figure 4a and b.

Supplementary Table S1 List of filtered variants identified through exome sequencing

CHROMOSOME	POSITION	GENE	dbSNP ID	REFERENCE ALLELE	ALTERNATIVE ALLELE	CODON	AA	EFFECT	DESCRIPTION	SIFT SCORE#
chr1	38034497	*GNL2*	rs781551158	T	C	cAg/cGg	Q608R	NON_SYNONYMOUS_CODING	guanine nucleotide binding protein-like 2 (nucleolar) (GNL2)	0.39
chr1	204124223	*REN*	None	A	G	cTg/cCg	L381P	NON_SYNONYMOUS_CODING	renin (REN)	0
chr1	247473097	*ZNF496*	rs759667017	C	A	Gcc/Tcc	A266S	NON_SYNONYMOUS_CODING	zinc finger protein 496 (ZNF496)	0.17
chr10	5436426	*TUBAL3*	None	T	G			INTRON	tubulin	None
chr10	30317782	*KIAA1462*	rs190116152	C	T	cGa/cAa	R432Q	NON_SYNONYMOUS_CODING	KIAA1462 (KIAA1462)	0
chr10	64022567	*RTKN2*	None	T	A	cAa/cTa	Q25L	NON_SYNONYMOUS_CODING	rhotekin 2 (RTKN2)	0.07
chr10	124812607	*ACADSB*	rs188094280	G	A	Gag/Aag	E387K	NON_SYNONYMOUS_CODING	acyl-CoA dehydrogenase	0.03
chr11	55653067	*SPRYD5*	rs755737200	T	G	Tgc/Ggc	C55G	NON_SYNONYMOUS_CODING	SPRY domain containing 5 (SPRYD5)	0
chr12	109994835	*MMAB*	None	A	G	Tga/Cga	*251R	STOP_LOST	methylmalonic aciduria (cobalamin deficiency) cblB type (MMAB)	None
chr12	124256153	*DNAH10*	rs200881150	C	T	Cgt/Tgt	R41C	NON_SYNONYMOUS_CODING	dynein	0.05
chr13	77718591	*MYCBP2*	None	C	T	gGc/gAc	G2431D	NON_SYNONYMOUS_CODING	MYC binding protein 2 (MYCBP2)	0.01
chr13	103384353	*CCDC168*	None	C	T	Gaa/Aaa	E6232K	NON_SYNONYMOUS_CODING	coiled-coil domain containing 168 (CCDC168)	0
chr13	109707861	*MYO16*	None	C	A	Caa/Aaa	Q1085K	NON_SYNONYMOUS_CODING	myosin XVI (MYO16)	None
chr17	1399169	*INPP5K*	rs758147788	G	A	cCg/cTg	P432L	NON_SYNONYMOUS_CODING	inositol polyphosphate-5-phosphatase K (INPP5K)	0.67
chr17	39658774	*KRT13*	None	G	A	Ctc/Ttc	L366F	NON_SYNONYMOUS_CODING	keratin 13 (KRT13)	0
chr17	40336194	*HCRT*	None	G	A	cCc/cTc	P125L	NON_SYNONYMOUS_CODING	hypocretin (orexin) neuropeptide precursor (HCRT)	0
chr17	47590234	*NGFR*	rs761987686	G	A	Gtt/Att	V383I	NON_SYNONYMOUS_CODING	nerve growth factor receptor (NGFR)	0.37
chr17	73998671	*CDK3*	rs779466474	T	C	aTt/aCt	I189T	NON_SYNONYMOUS_CODING	cyclin-dependent kinase 3 (CDK3)	0.06
chr19	38028084	*ZNF793*	rs200312043	G	A	cGt/cAt	R175H	NON_SYNONYMOUS_CODING	zinc finger protein 793 (ZNF793)	0.29
chr19	40411829	*FCGBP*	rs147081983	G	A	Cgc/Tgc	R1267C	NON_SYNONYMOUS_CODING	Fc fragment of IgG binding protein (FCGBP)	0.18
chr19	51871803	*CLDND2*	rs765336897	C	G	gGg/gCg	G10A	NON_SYNONYMOUS_CODING	claudin domain containing 2 (CLDND2)	0.23
chr2	10559956	*HPCAL1*	rs769646547	C	A	Cac/Aac	H25N	NON_SYNONYMOUS_CODING	hippocalcin-like 1 (HPCAL1)	None
chr2	28796168	*PLB1*	rs776639920	G	C	Gct/Cct	A477P	NON_SYNONYMOUS_CODING	phospholipase B1 (PLB1)	None
chr2	55435804	*C2orf63*	rs755855981	C	T	cGc/cAc	R286H	NON_SYNONYMOUS_CODING	chromosome 2 open reading frame 63 (C2orf63)	0.26
chr2	55812231	*SMEK2*	rs1454257382	T	C	Atg/Gtg	M397V	NON_SYNONYMOUS_CODING	SMEK homolog 2	0.35
chr2	55900149	*PNPT1*	rs774197508	C	T	Gtg/Atg	V249M	NON_SYNONYMOUS_CODING	polyribonucleotide nucleotidyltransferase 1 (PNPT1)	0.04
chr2	73519603	*EGR4*	None	T	C	gAt/gGt	D251G	NON_SYNONYMOUS_CODING	early growth response 4 (EGR4)	0.32
chr2	74777365	*LOXL3*	None	C	T	Gtc/Atc	V142I	NON_SYNONYMOUS_CODING	lysyl oxidase-like 3 (LOXL3)	0.02
chr22	19482035	*CDC45*	rs151216373	C	G	tCt/tGt	S183C	NON_SYNONYMOUS_CODING	cell division cycle 45 homolog (S. cerevisiae) (CDC45)	0.04
chr22	29736735	*AP1B1*	None	G	T	gaC/gaA	D636E	NON_SYNONYMOUS_CODING	adaptor-related protein complex 1	0.05
chr22	32108801	*PRR14L*	rs750572033	G	T	gCa/gAa	A1675E	NON_SYNONYMOUS_CODING	proline rich 14-like (PRR14L)	None
chr22	38690399	*CSNK1E*	rs369141815	T	C	Agt/Ggt	S343G	NON_SYNONYMOUS_CODING	casein kinase 1	0.74
chr22	43976471	*EFCAB6*	None	T	C	aAg/aGg	K1034R	NON_SYNONYMOUS_CODING	EF-hand calcium binding domain 6 (EFCAB6)	0.58
chr3	66431945	*LRIG1*	rs761627996	C	T	Gcg/Acg	A910T	NON_SYNONYMOUS_CODING	leucine-rich repeats and immunoglobulin-like domains 1 (LRIG1)	0.63
chr3	100277249	*TMEM45A*	rs144916545	C	G	gCc/gGc	A135G	NON_SYNONYMOUS_CODING	transmembrane protein 45A (TMEM45A)	None
chr3	127441392	*MGLL*	rs201772034	C	T	Gaa/Aaa	E94K	NON_SYNONYMOUS_CODING	monoglyceride lipase (MGLL)	0.34
chr3	134323301	*KY*	rs757640041	G	A	aCg/aTg	T369M	NON_SYNONYMOUS_CODING	kyphoscoliosis peptidase (KY)	0.2
chr3	136016892	*PCCB*	rs201984177	G	A	Gtc/Atc	V308I	NON_SYNONYMOUS_CODING	propionyl CoA carboxylase	0.41
chr3	150128593	*TSC22D2*	rs776118078	A	G	Atg/Gtg	M486V	NON_SYNONYMOUS_CODING	TSC22 domain family	0.43
chr3	182871951	*LAMP3*	None	T	G	aAc/aCc	N93T	NON_SYNONYMOUS_CODING	lysosomal-associated membrane protein 3 (LAMP3)	0.37
chr4	48990620	*CWH43*	None	T	G	aTt/aGt	I57S	NON_SYNONYMOUS_CODING	cell wall biogenesis 43 C-terminal homolog (S. cerevisiae) (CWH43)	None
chr4	106552093	*ARHGEF38*	rs975793457	A	T	Att/Ttt	I183F	NON_SYNONYMOUS_CODING	Rho guanine nucleotide exchange factor (GEF) 38 (ARHGEF38)	0
chr5	156186367	*SGCD*	rs397516337	C	T	tCc/tTc	S280F	NON_SYNONYMOUS_CODING	sarcoglycan	0.12
chr5	167675335	*TENM2*	rs182071305	A	G	aAt/aGt	N2455S	NON_SYNONYMOUS_CODING	odz	0.08
chr6	119243175	*MCM9*	None	T	A	aAa/aTa	K233I	NON_SYNONYMOUS_CODING	minichromosome maintenance complex component 9 (MCM9)	0.02
chr6	160454090	*IGF2R*	rs766496292	G	A	Gat/Aat	D388N	NON_SYNONYMOUS_CODING	insulin-like growth factor 2 receptor (IGF2R)	0.6
chr7	6193974	*USP42*	rs1038602703	C	T	cCg/cTg	P930L	NON_SYNONYMOUS_CODING	ubiquitin specific peptidase 42 (USP42)	0.36
chr8	11142438	*MTMR9*	rs141460029	A	G	aAt/aGt	N14S	NON_SYNONYMOUS_CODING	myotubularin related protein 9 (MTMR9)	None
chr9	72130936	*APBA1*	None	C	T	atG/atA	M397I	NON_SYNONYMOUS_CODING	amyloid beta (A4) precursor protein-binding	None

#With a SIFT score smaller than 0.05 the corresponding variant is predicted as 'Damaging', otherwise it is predicted as 'Tolerated'.
